# Identification of potential therapeutic drugs targeting core genes for systemic lupus erythematosus (SLE) and coexisting COVID‐19: Insights from bioinformatic analyses

**DOI:** 10.1002/iid3.1087

**Published:** 2023-11-20

**Authors:** Chao Chen, Hongjian Zhang, Yanbin Lin, Meiqi Lu, Quan Liao, Shichao Zhang, Weibin Chen, Xiongwei Zheng, Yunpeng Li, Rui Ding, Zheng Wan

**Affiliations:** ^1^ School of Medicine, Institute of Genomics Huaqiao University Xiamen China; ^2^ Department of Oncology and Vascular Interventional Radiology Zhongshan Hospital Xiamen University, School of Medicine, Xiamen University Xiamen Fujian China

**Keywords:** bioinformatic, differentially expressed genes, severe coronavirus disease 2019 (COVID‐19), systemic lupus erythematosus (SLE), targeted drugs

## Abstract

**Objective:**

Systemic lupus erythematosus (SLE) patients are at risk during the COVID‐19 pandemic, yet the underlying molecular mechanisms remain incompletely understood. This study sought to analyze the potential molecular connections between COVID‐19 and SLE, employing a bioinformatics approach to identify effective drugs for both conditions.

**Methods:**

The data sets GSE100163 and GSE183071 were utilized to determine share differentially expressed genes (DEGs). These DEGs were later analyzed by various bioinformatic methods, including functional enrichment, protein–protein interaction (PPI) network analysis, regulatory network construction, and gene–drug interaction construction.

**Results:**

A total of 50 common DEGs were found between COVID‐19 and SLE. Gene ontology (GO) functional annotation revealed that “immune response,” “innate immune response,” “plasma membrane,” and “protein binding” were most enriched in. Additionally, the pathways that were enriched include “Th1 and Th2 cell differentiation.” The study identified 48 genes/nodes enriched with 292 edges in the PPI network, of which the top 10 hub genes were CD4, IL7R, CD3E, CD5, CD247, KLRB1, CD40LG, CD7, CR2, and GZMK. Furthermore, the study found 48 transcription factors and 8 microRNAs regulating these hub genes. Finally, four drugs namely ibalizumab (targeted to CD4), blinatumomab (targeted to CD3E), muromonab‐CD3 (targeted to CD3E), and catumaxomab (targeted to CD3E) were found in gene–drug interaction.

**Conclusion:**

Four possible drugs that targeted two specific genes, which may be beneficial for COVID‐19 patients with SLE.

## Graphical abstract

(1) This study sought to analyze the potential molecular connections between COVID‐19 and systemic lupus erythematosus (SLE), employing a bioinformatics approach to identify effective drugs for both conditions. (2) Four drugs namely ibalizumab (targeted to CD4), blinatumomab (targeted to CD3E), muromonab‐CD3 (targeted to CD3E), and catumaxomab (targeted to CD3E) were found. (3) Four possible drugs that targeted two specific genes, which may be beneficial for COVID‐19 patients with SLE.

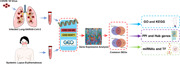

## 1 INTRODUCTION

The COVID‐19 pandemic has resulted in high mortality rates, caused by the severe acute respiratory syndrome coronavirus 2 (SARS‐CoV‐2), causing a worldwide disaster, with over 6.9 million deaths recorded as of May 2023.^1^ COVID‐19 is known to affect multiple organ systems such as the cardiovascular, respiratory, digestive, and immune systems, making it a systemic disease.^2^ COVID‐19 can induce the production of pro‐inflammatory cytokines that may result in cytokine dysregulation,^3^ which poses a significant risk for individuals with underlying conditions, such as systemic lupus erythematosus (SLE) patients.

SLE is a multifaceted autoimmune disorder in which the immune system aberrantly targets the body's own tissues and organs.^4^ This disorder can manifest across diverse physiological systems, notably the hematological, musculoskeletal, and central nervous systems. During the COVID‐19 pandemic, individuals with SLE encounter distinct vulnerabilities. Primarily, the systemic inflammation elicited by COVID‐19^5^ may exacerbate the clinical manifestations of SLE, encompassing articular discomfort, cutaneous presentations, nephritic inflammation, and cardiovascular anomalies. Further, an inherent immunological fragility characterizes these patients, predisposing them to immune dysregulation.^6^ COVID‐19 can further disrupt their compromised immune system, leading to intensified inflammation and increased risk of disease exacerbation.^7^ Moreover, the management of SLE patients during the pandemic is challenging, as treatment modifications to minimize infection risk may impact disease control.^8^ Therefore, it is urgent to find new effective treatments for SLE patients during the COVID‐19 pandemic.

**Figure 1:**
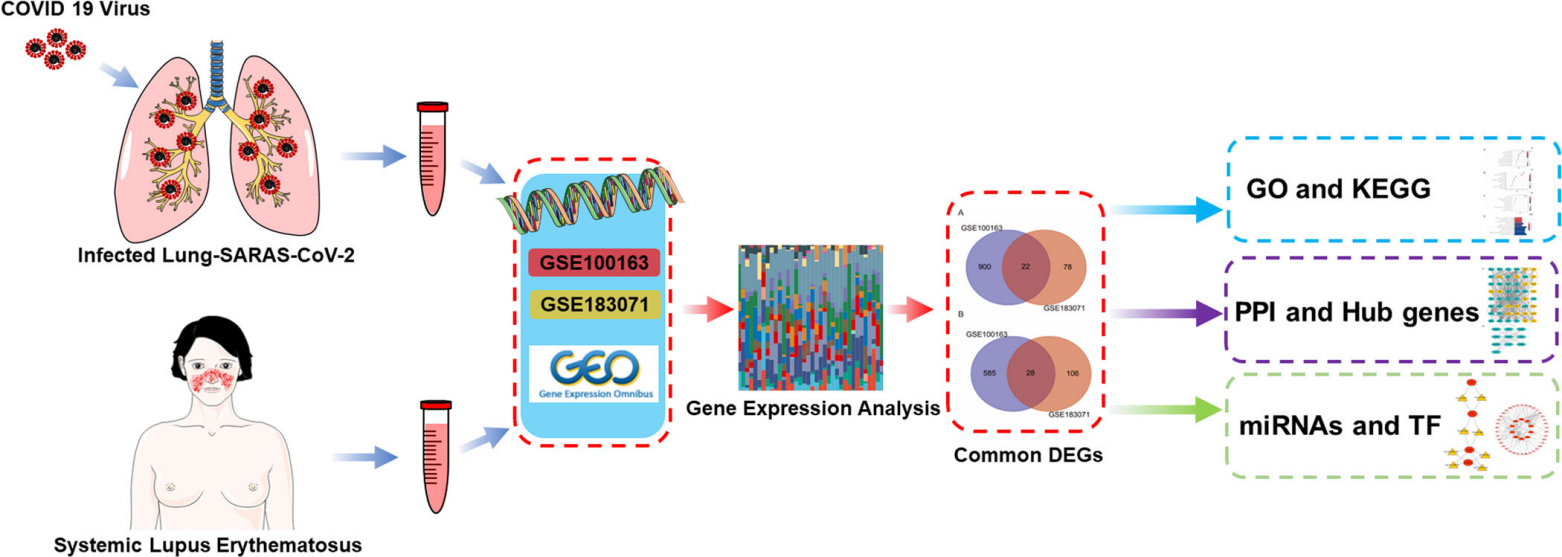
The workflow of this study.

The information provided above represents observed phenomenon without definitive conclusions, and further research is needed to understand their true association. The differentially expressed gene (DEGs) between the patients and the healthy are the key to know diseases. Bio‐health researchers are actively using bioinformatics methods to screen DEGs to diagnose, classify, and predict diseases, including cancers,^9^, ^10^ and cardiovascular disease.^11^ To elucidate the mechanistic interplay between COVID‐19 infections and SLE, we employed an exhaustive bioinformatics analytical approach. Initially, DEGs were identified from two data sets: GSE100163, which encompasses SLE samples, and GSE183071, which pertains to COVID samples. Subsequently, DEGs common to both data sets were pinpointed. This was followed by comprehensive analyses encompassing functional enrichment and pathway exploration. Moreover, a protein–protein interaction (PPI) network was delineated, and central hub genes were determined. In the concluding stages, potential therapeutic agents and transcriptional modulators were identified. Figure 1 shows the workflow of this study.

## 2 MATERIALS AND METHODS

### 2.1 Data collection

GSE100163 contains 55 SLE blood samples and 14 normal samples. The platform is GPL6884 (Illumina HumanWG‐6 v3.0). GSE183071 contains 35 COVID‐19 blood samples and 19 normal samples. The platform is GPL30569 (NanoString nCounter GX Human Immunology v2).

### 2.2 Data processing

The online tool GEO2R (https://www.ncbi.nlm.nih.gov/geo/geo2r/)^12^ was utilized to acquire the DEGs. The criteria for DEGs were set at |logFC| > 1 and *p* < .05. A logFC > 1 indicates upregulated gene, while a logFC < −1 indicates downregulated gene. The common DEGs were determined using a Venn diagram.

### 2.3 Gene ontology (GO) and Kyoto encyclopedia of genes and genomes (KEGG) pathway enrichment analysis

The DAVID 6.8 database (http://david.abcc.ncifcrf.gov/) was used for GO and KEGG pathway analysis.^13^ A *p* value of less than .05 was considered significant.

### 2.4 PPI network analysis and hub gene extraction

To identify the interactions between DEGs, the study utilized the STRING database,^14^ available at http://string-db.org. The Cytoscape software (v3.7.1) was used to analyze and visualize the PPI network. The top 10 genes were identified using the cytoHubba plugin. CytoHubba offers 11 different topological analysis methods. Among these methods, MCC stands out for its superior precision in predicting essential proteins within the PPI network.^15^

### 2.5 Recognition of hub genes associated transcription factors (TFs) and microRNA (miRNA)

We used the NetworkAnalyst platform^16^ and mirTarbase^17^ to find hub genes associated with TFs and miRNA.

### 2.6 Evaluation of applicant drugs

The DGIDB database (https://dgidb.genome.wustl.edu/) was used to identify existing drugs or compounds that could potentially targeted to hub genes.^18^

## 3 RESULTS

### 3.1 Identification of DEGs

**Figure 2:**
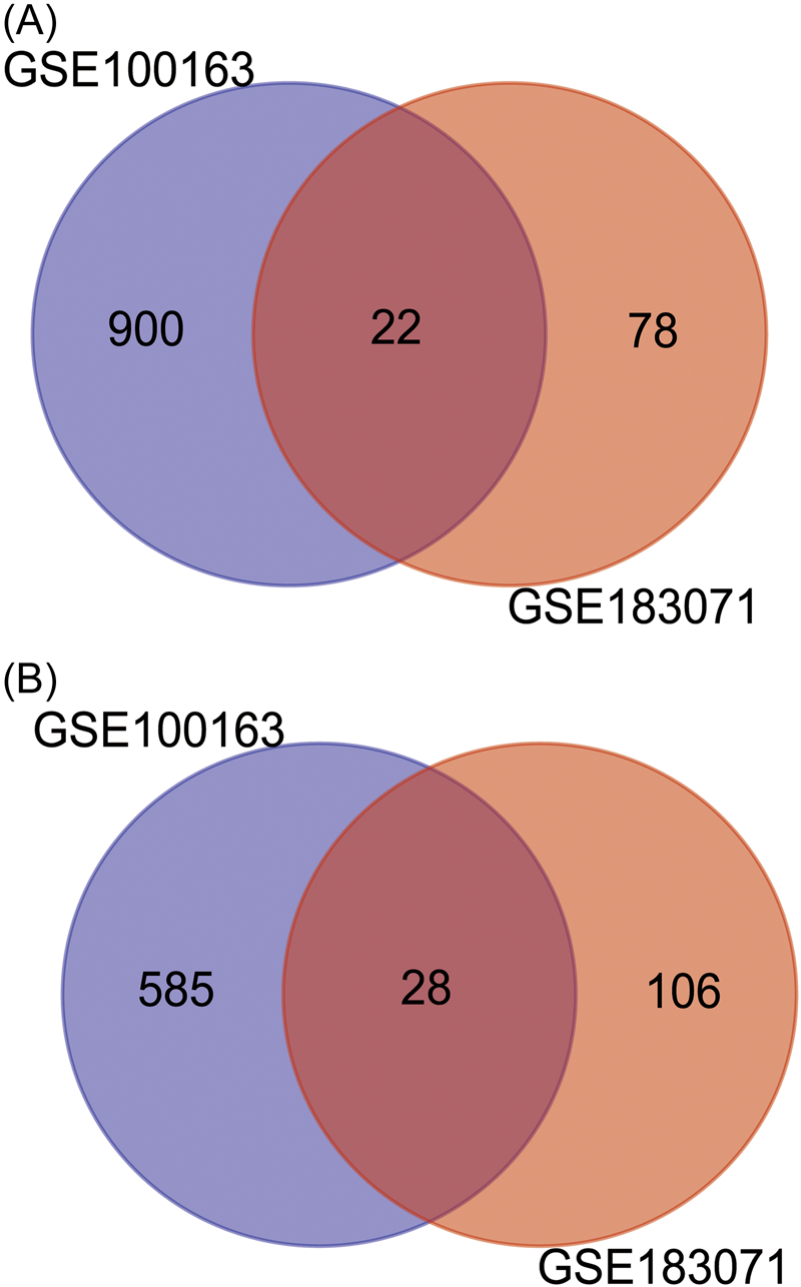
Common differentially expressed genes (DEGs) identification for COVID‐19 patients and systemic lupus erythematosus (SLE). (A) 22 common DEGs with upregulation between COVID‐19 patients with SLE. (B) 28 common DEGs with downregulation between COVID‐19 patients with SLE.

DEGs, consisting of 1535 for GSE100163 and 234 for GSE183071, were identified. Further comparison of the two data sets revealed an overlap of 50 genes, as shown in Figure 2. From these shared DEGs, 22 were upregulated and 28 were downregulated (Table 1).

**Table 1:** The summary of differentially expressed genes (DEGs).

DEGs	Gene name
Upregulated genes	TICAM1, PML, BST2, CLEC4E, TNFSF15, ATG10, LTF, PRKCD, IL1RN, TNFAIP6, MAPK14, JAK2, ATG7, ARG1, TLR5, CAMP, NOD2, IFITM1, IL18R1, LILRA5, BATF, LTB4R
Downregulated genes	RPL19, CD4, SH2D1A, GPR183, DPP4, KLRC3, STAT4, ETS1, CD247
	GZMK, ABCB1, CD5, IL7R, CD96, CR2, FCER1A, HLA‐DMB, CD7, NT5E, TCF7, IL1RAP, CSF1R, TGFBI, CD3E, CD40LG, EEF1G, KLRB1, KLRF1

### 3.2 GO and KEGG pathway enrichment analysis

**Figure 3:**
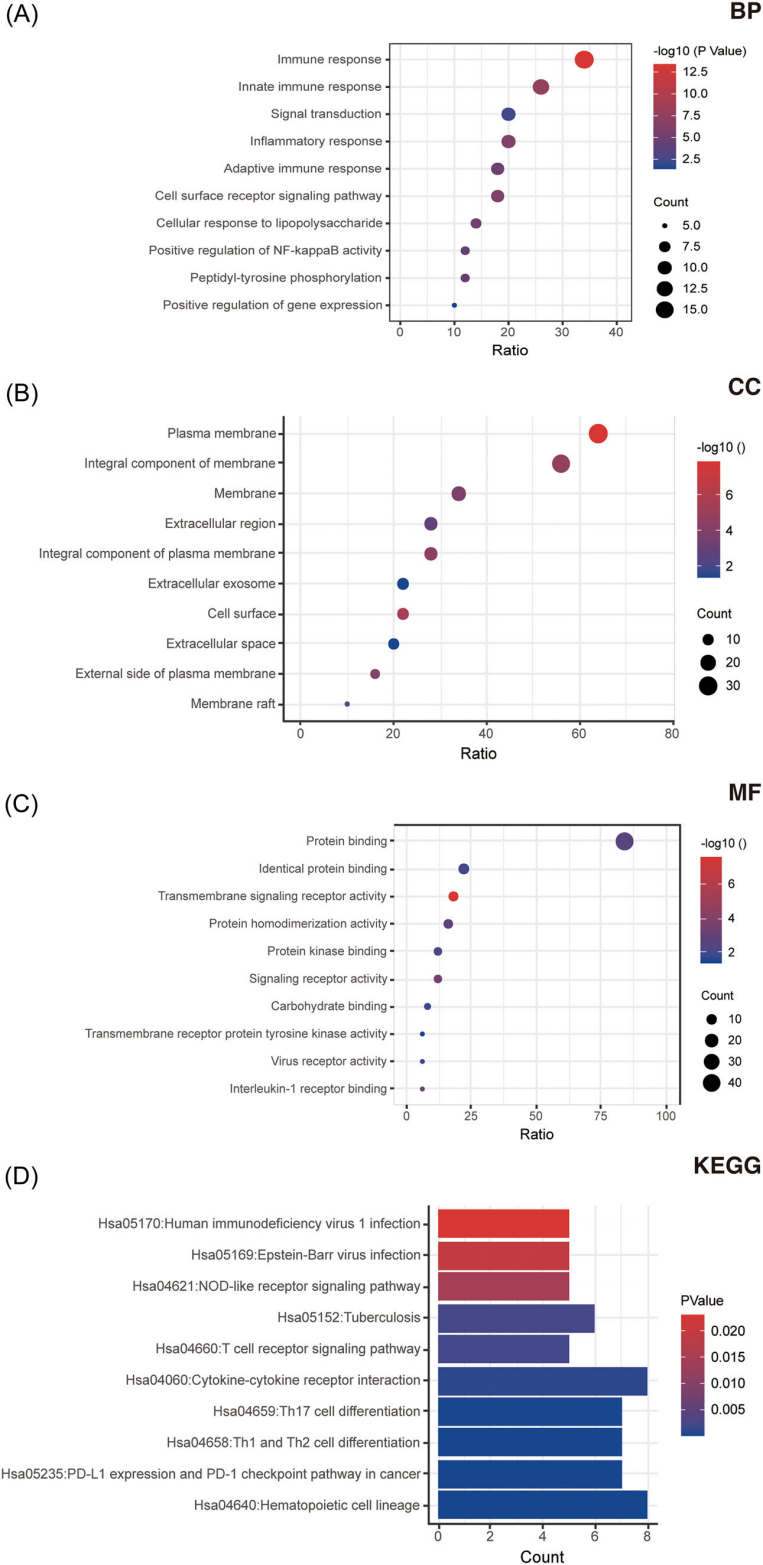
Functional analyses including biological process (BP), molecular function (MF), and cellular component (CC), and KEGG pathway analyses, separately.

The GO annotation based on biological process revealed enrichment in categories such as “innate immune response,” “inflammatory response,” and “adaptive immune response.” In terms of cellular component (CC), the analysis identified enrichment in the “integral component of membrane,” “membrane,” and “integral component of plasma membrane.” The molecular function (MF) annotation analysis showed in “identical protein binding,” “protein binding,” and “protein kinase binding.” Moreover, the pathway analysis showed enrichment in “Th1 and Th2 cell differentiation,” “human immunodeficiency virus (HIV) 1 infection,” and “Th17 cell differentiation,” according to KEGG pathway analysis (Figure 3).

### 3.3 The construction of PPI and hub genes analysis

**Figure 4:**
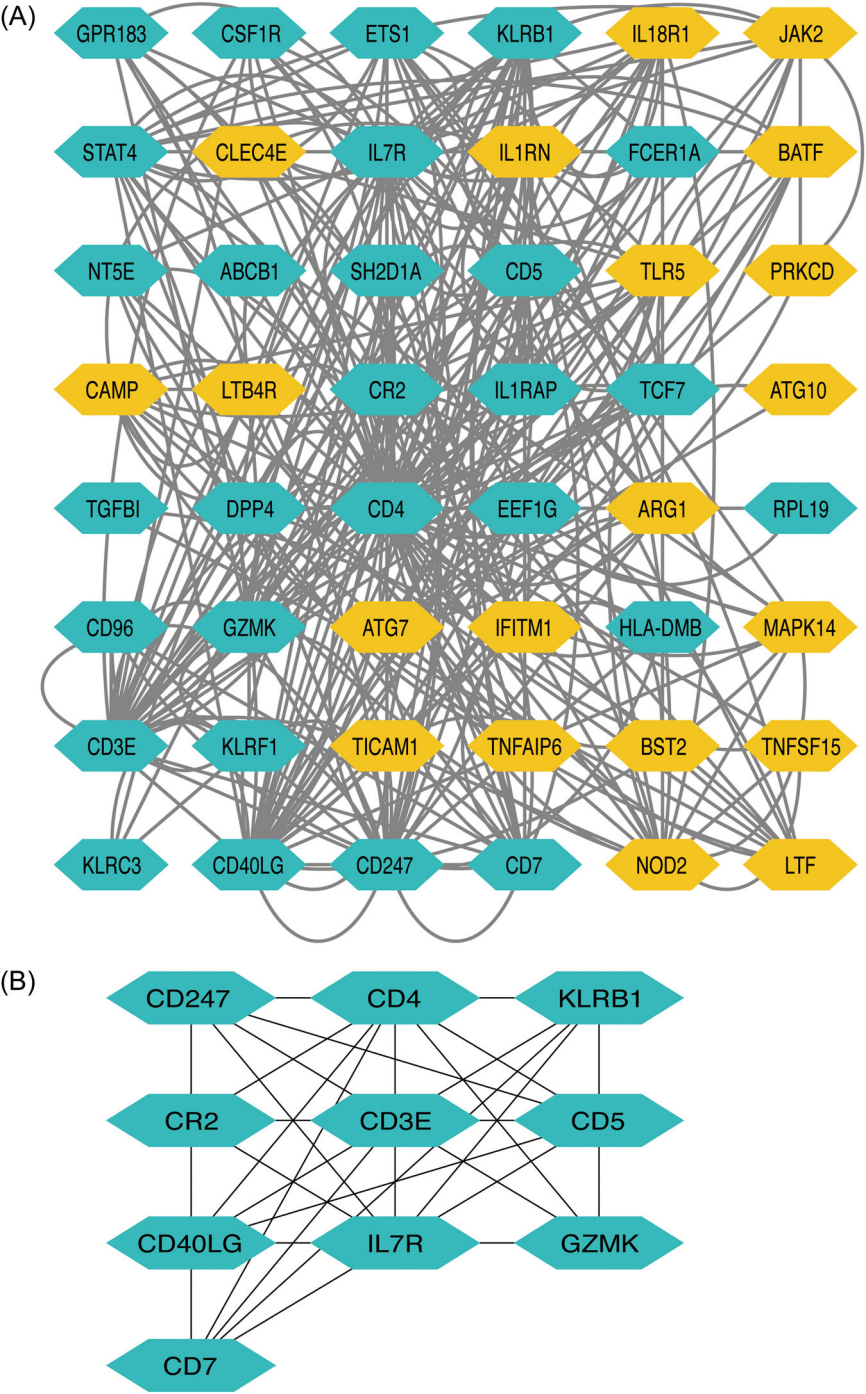
Protein–protein interaction network (A) and hub genes (B). Yellow ellipses stand for upregulation gene, while blue ellipses for downregulation.

We revealed that 48 genes/nodes with 292 edges (Figure 4A). The top 10 hub genes were CD4, CD7, CD40LG, CD3E, CD247, CR2, GZMK, IL7R, KLRB1, and KLRD1 (Figure 4B).

### 3.4 Construction of regulatory networks

**Figure 5:**
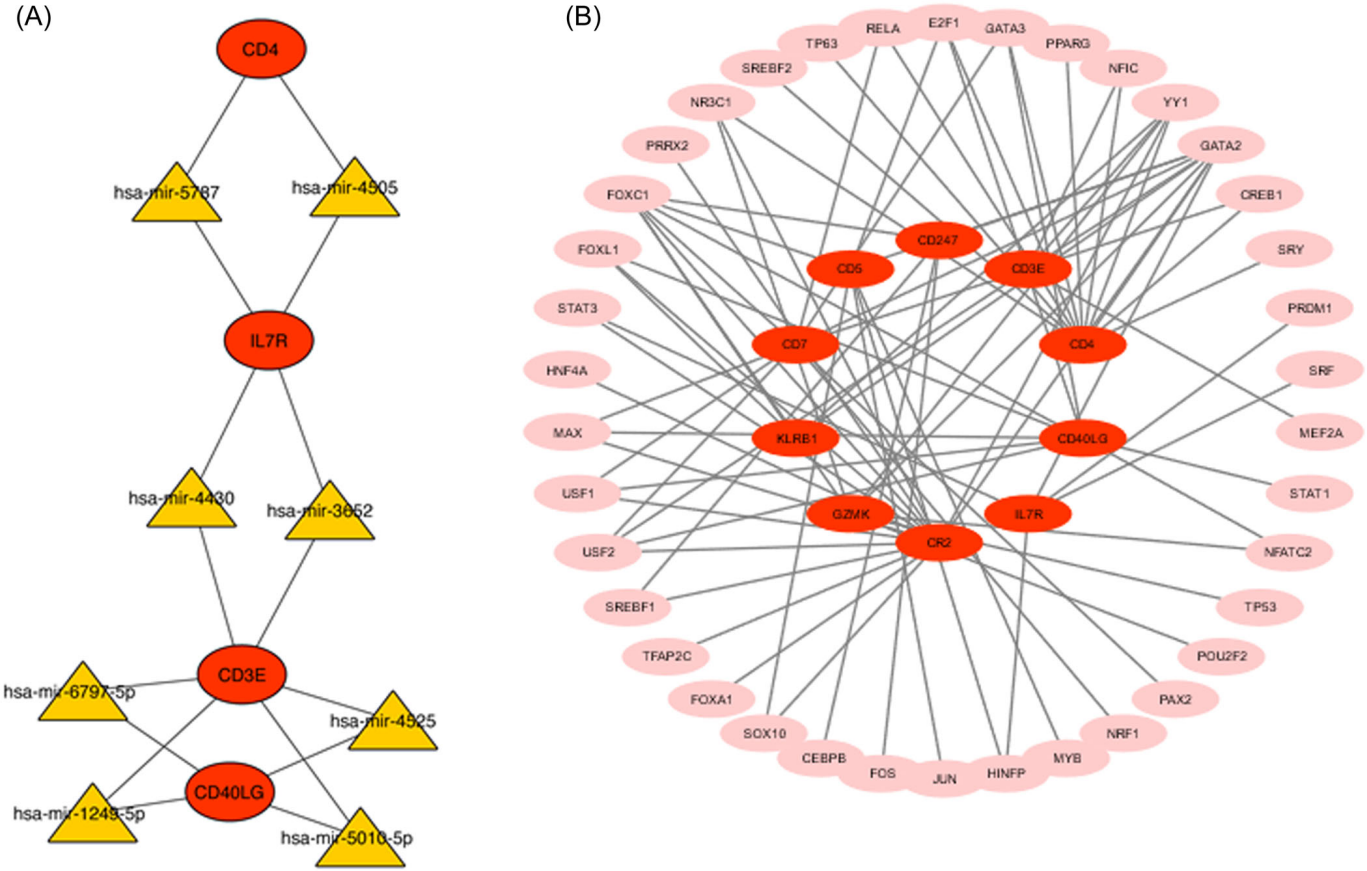
(A) MicroRNAs (miRNAs) interactions with hub genes. Red ellipses represent hub genes, while yellow represents miRNAs. (B) The hub genes and transcription factors (TFs) interaction network. Pink represents miRNAs, red indicates hub genes.

We analyzed the regulatory interaction network between TF and microRNA genes and identified 48 TFs genes and eight miRNAs genes that regulate hub genes. This finding indicates significant interference between the two regulatory signals. The interaction between regulatory miRNAs and hub genes is illustrated in Figure 5A, whereas Figure 5B illustrates the interaction between hub genes and TFs.

### 3.5 The construction of drug–gene interaction

The drugs in the DGIDB database, including ibalizumab (targeted to CD4), blinatumomab (targeted to CD3E), muromonab‐cd3(targeted to CD3E), catumaxomab (targeted to CD3E). (Table 2).

**Table 2:** The candidate drugs and corresponding genes for COVID‐19 and SLE.

Gene	Drug	Interaction
CD4	IBALIZUMAB	inhibitor
CD3E	BLINATUMOMAB	antibody
CD3E	MUROMONAB‐CD3	inhibitor
CD3E	CATUMAXOMAB	antibody

Abbreviation: SLE, systemic lupus erythematosus.

## 4 DISCUSSION

The COVID‐19 pandemic has resulted in high mortality rates for patients with underlying conditions,^1^ such as SLE. Although some reports found the phenomenon that SLE patients are associated with an increased viral susceptibility, leading to severe COVID‐19,^7^, ^8^ but the molecular mechanisms between SLE and COVID‐19 are still unknown. This study was to investigate the molecular mechanisms and identify potential targeted drugs for SLE patients who have contracted COVID‐19.

We analyzed two data sets from the GEO database and identified 50 common DEGs. These DEGs demonstrated the same expression trend in SLE and COVID‐19. Via GO enrichment analysis, the DEGs were found to have functions related to the immune system, including “immune response,” among others. In KEGG pathways, they were involved in immune‐related pathways. SLE patients have a higher susceptibility to severe COVID‐19, and if infected, they may experience a more severe clinical course due to SLE‐related innate immune disorders and increased baseline inflammation.^7^ Using Cytoscape, we analyzed the PPI network and screened out 10 important hub genes, including CD4, CD7, CD40LG, CD3E, CD247, CR2, GZMK, IL7R, KLRB1, and KLRD1. After gene–drug analysis, four drugs namely ibalizumab (targeted to CD4), blinatumomab (targeted to CD3E), muromonab‐CD3 (targeted to CD3E), and catumaxomab (targeted to CD3E) were found.

The characteristic of SLE is the excessive activity of B cells to produce autoantibodies,^19^ but T‐cells also play a crucial role in the development of SLE. Helper T cells express CD4, the primary marker, which binds to the nonpolypeptide region of the major histocompatibility complex II and participates in antigen recognition in the helper T cell TCR.^20^ CD4+ T cells produce various cytokines, express costimulatory ligands, enhance CD8+ T cell function, promote B‐cell antibody production, and facilitate the destruction of abnormal cells, contributing to the innate immune response.^21^ Several viruses, such as Dengue virus,^22^ HIV virus,^23^ and influenza virus,^24^ have also been associated with CD4+ T cells' protective effects in infections. Our research revealed that CD4 expression was inhibited in both SLE and COVID‐19, consistent with earlier studies. This indicates the integral role of CD4 in immune disorders for both diseases. CD3E codes for CD3‐epsilon polypeptides, which with CD3‐gamma, ‐delta, ‐zeta, and somatic cell receptors form TCR–CD3 complexes and play a significant role in antigen recognition and intracellular signaling pathways.^25^ Our research indicates that CD3E and CD4 show consistent expression trends in SLE, making them key genes.

Monoclonal antibodies (mAbs) are named after the clones of white cells responsible for their production. These antibodies have gained significant attention recently because of their diverse applications in fields such as medicine and biotechnology.^26^ They have been used successfully to develop drugs for the prevention and treatment of viral infections such as HIV.^27^ However, there is still no effective solution for the treatment of SLE in COVID‐19 patients. In our drug targeting prediction results, four important drugs were obtained: ibalizumab (targeted to CD4), blinatumomab, muromonab‐cd3, catumaxomab (targeted to CD3E). They may be an important targeted drug for patients with COVID‐19 and SLE.

Ibalizumab, an approved treatment for HIV‐1 infection, functions as a CD4‐directed postattachment inhibitor.^28^ Its primary role is to hinder the entry of HIV‐1 into CD4 cells,^29^ while still preserving normal immune function. Notably, as a CD4‐targeted drug, it possesses robust safety features and exhibits efficacy against drug resistance.^30^ This makes it the first mAb employed for managing multidrug‐resistant (MDR) HIV‐1 infection.^31^ No data exist on the use of this medication in SLE patients with COVID‐19. Hence, further research and development are necessary to establish its specific indication for the treatment of these patients.

Blinatumomab is a bispecific antibody approved to treat refractory B‐cell precursor acute lymphoblastic leukemia (ALL).^32^ It targets CD19 and T cell CD3 to connect T cells and cancer cells, bringing T cells close to the cancer cells and achieving a killing effect.^33^ Furthermore, Blinatumomab binding to T cells can activate the T cell signaling pathway, promoting T cell proliferation.^34^ Muromonab‐CD3 is the first mAb approved for renal transplantation.^35^ It targets the CD3 antigen, which is present in all mature human T cells, and reacts with T cells only from higher primates such as chimpanzees, not with other human tissues. It belongs to the immunoglobulin two‐immunoglobulin class and binds to a subunit of the CD3 complex, which is part of the T‐cell receptor. By doing so, it blocks the function of the adjacent complex that recognizes foreign antigens during T cell recognition.^36^ Catumaxomab, the first dual antibody that recruits T cells,^37^ has been approved by the US Food and Drug Administration and has demonstrated promising efficacy in treating ovarian and other ascites‐related cancers.

Our research also investigated the relationship between TFs and miRNAs in the diseases. We identified 48 TFs and eight miRNAs that regulate hub genes. Among the miRNAs we obtained, has‐miR‐4505 was detected in influenza virus.^38^ However, the role of these regulators in COVID‐19 and SLE needs to be further explored.

Our study proffers innovative perspectives on the molecular interconnections between SLE and COVID‐19, concurrently proposing potential avenues for therapeutic interventions. Nonetheless, it is crucial to critically consider a series of intrinsic limitations inherent to our research design and methodology. First, the foundational results of our investigation are primarily derived from the analysis of two distinct data sets retrieved from the GEO database, rendering our findings predominantly observational. While we have successfully identified a subset of DEGs manifesting consistent expression patterns across both SLE and COVID‐19 conditions, these observations do not provide a sufficient basis to unequivocally establish causation or directly delineate the mechanistic pathways linking these specific genes to an augmented susceptibility or heightened severity of COVID‐19 in patients with SLE. Second, the potential therapeutic compounds identified, inclusive of ibalizumab, blinatumomab, muromonab‐CD3, and catumaxomab, exhibit associations with the pertinent genes under investigation. However, it is imperative to recognize that these associations may not extend uniformly across the entire spectrum of SLE and COVID‐19 patients. The inherent variability in gene expression profiles and underlying pathophysiological mechanisms across individuals underscores the imperative for comprehensive cohort studies, aimed at validating the widespread applicability and relevance of our findings.

## 5 CONCLUSION

We identified four drugs that target two crucial genes for both COVID‐19 and SLE. These drugs may provide benefits for individuals with SLE who are also suffering from COVID‐19.

## AUTHOR CONTRIBUTIONS

Chao Chen and Zheng Wan were contributed to the study conception and design. Material preparation, data collection and analysis were performed by Hongjian Zhang, Yanbin Lin, Meiqi Lu, Quan Liao, Shichao Zhang, Weibin Chen, Xiongwei Zheng, Yunpeng Li, and Rui Ding. The first draft of the manuscript was written by Chao Chen, Hongjian Zhang, Yanbin Lin, Meiqi Lu, and Zheng Wan. All authors read and approved the final manuscript.

## CONFLICT OF INTEREST STATEMENT

The authors declare no conflict of interest.

## ETHICS STATEMENT

The data used in the paper are from the open and public GEO database, which is not need to permit by local ethics committee.
